# Identification of Biomarkers Associated with Cancerous Change in Oral Leukoplakia Based on Integrated Transcriptome Analysis

**DOI:** 10.1155/2022/4599305

**Published:** 2022-01-19

**Authors:** Chunshen Li, Yingying Shi, Lihua Zuo, Mingzhe Xin, Xiaomeng Guo, Jianli Sun, Shuai Chen, Bin Zhao, Zhe Yang, Zhi Sun, Hongyu Zhao

**Affiliations:** ^1^Department of Oral Emergency, The First Affiliated Hospital of Zhengzhou University· Stomatological Hospital of Henan Province, Zhengzhou 450000, China; ^2^School and Hospital of Stomatology of Zhengzhou University, Zhengzhou 450000, China; ^3^Department of Pharmacy, The First Affiliated Hospital of Zhengzhou University, Zhengzhou 450000, China; ^4^Henan Engineering Research Center of Clinical Mass Spectrometry for Precision Medicine, Zhengzhou 450000, China; ^5^Department of Oral and Maxillofacial Surgery, The First Affiliated Hospital of Zhengzhou University·Stomatological Hospital of Henan Province, Zhengzhou 450000, China

## Abstract

**Objective:**

Oral leukoplakia (OLK) is the most common precancerous lesion in the oral cavity. This study aimed to explore key biomarkers for monitoring OLK for early diagnosis of oral squamous cell carcinoma (OSCC) and screen small-molecule drugs for the prevention of OSCC.

**Method:**

The Gene Expression Omnibus (GEO) database was explored to extract two microarray datasets, namely, GSE85195 and GSE25099. The data of the normal group, OLK group, and OSCC group were analyzed by weighted gene coexpression network analysis (WGCNA) to identify the most significant gene module and differentially expressed genes (DEGs). The intersection genes were extracted as the key genes of OLK carcinogenesis. Subsequently, Gene Ontology (GO) and Kyoto Encyclopedia of Genes and Genomes (KEGG) pathways were analyzed in the module. Connectivity Map and molecular docking were used to screen small-molecule drugs. The diagnostic values of four key genes were identified and verified in the GSE26549 dataset.

**Results:**

WGCNA obtained the red module (*r* = −0.91, *p* < 0.05) with the strongest correlation with cancerous phenotype. GO enrichment analysis showed 60 pathways, including 28 biological processes, 11 cell components, and 21 molecular functions, and KEGG enrichment analysis showed 4 pathways (*p* < 0.05). In the differential expression analysis, there was no intersection between the upregulated genes and the red module genes. However, the intersection of the downregulated genes and the red module genes yielded 4 key genes: dopachrome tautomerase (DCT), keratin 3 (KRT3), keratin 76 (KRT76), and FAM3 metabolic regulation signal molecule B (FAM3B). The area under the curve of the diagnostic model constructed by these four genes was 0.963 (CI = 0.913–1.000). The sensitivity was 0.933, and the specificity was 0.923. The diagnostic model was successfully verified in GSE26549 (AUC = 0.745, CI = 0.638–0.851). Compared with the diagnostic models of the previous studies, the diagnostic efficiency of this model was the highest. The small-molecule drugs, selumetinib and benidipine, were selected according to the gene expression profile and showed binding activity when docking with the above molecules.

**Conclusions:**

This study provides new targets and drugs for OLK. These targets could be used as the key diagnostic molecules for long-term follow-up of OLK. The small-molecule drugs selumetinib and benidipine could be used for the prevention and treatment of OSCC.

## 1. Introduction

Oral leukoplakia (OLK) is a potentially malignant disease, with an incidence of 409.2 per 100,000 persons in men and 70.0 in women [1]. The pathological manifestations of OLK are various degrees of abnormal epithelial hyperplasia, which eventually progresses to malignant transformation and invades the surrounding tissues [2]. OLK is the most common precancerous lesion in the oral cavity. The malignant transformation rate is between 1% and 40% [3]. Clinically, photodynamic therapy, microwave therapy, and surgical resection are usually used to prevent cancer development [4]. The clinical manifestations and pathology of patients need to be followed up and monitored. Currently, there is no specific drug for oral squamous cell carcinoma (OSCC) treatment. Therefore, early diagnosis is an important means to reduce morbidity and mortality and improve prognosis. Due to the insidious onset and nonspecific symptoms of OLK, the diagnosis of OLK is often delayed. It is well known that changes in gene expression usually precede histopathological changes and are closely associated with the progression of cancers [5]. Therefore, abnormal gene expression has become a new perspective for the early diagnosis of OLK.

Currently, there are no clear diagnostic methods to predict whether OLK would become cancer. Cai et al. [6] found that SPP1 increased as normal tissues progressed to OLK and OSCC tissues. Fernanda Herrera Costa et al. [7] found that ALDH1A1 was positive in OLK tissues and negative in OSCC tissues, while ALDH1A2 was negative in OLK tissues and positive in OSCC tissues, which can be used as potential biomarkers for early detection of OSCC. However, this method only focused on the effect of the single gene, which cannot reveal the genetic relationship and build the relationship between genes and diseases. With the development of bioinformatics, large-sample, high-throughput gene microarray chip sequencing technology is increasingly used for the screening of diagnostic biomarkers. The most important bioinformatic analysis method is weighted gene coexpression network analysis (WGCNA). WGCNA is a method of constructing an expression network by using correlations between genes to mine gene modules that are highly related to external biological traits. It is highly sensitive to genes with small fluctuations and magnifies potential organisms in functional enrichment research. The signal has been successfully used in the analysis of a variety of diseases. Niemira et al. [8] used WGCNA to identify 4 new molecular targets related to nonsmall cell lung cancer, to gain an understanding of the pathogenesis of the disease. Yang et al. [9] used WGCNA to construct a four-gene prognostic model for liver cancer, and its C-index has better predictive potential than TNM staging.

**Figure 1:**
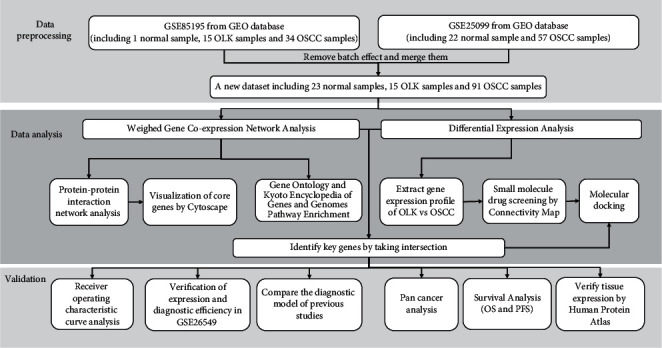
Flow chart of this research.

In this study, the combined mRNA sequencing data of multiple microarray chips were used for WGCNA and differential expression analysis, pathway enrichment analysis, and protein interaction network construction to obtain key genes and pathways for OLK carcinogenesis. The results were used in centralized validation of external data. We also screened small-molecule drugs that could prevent OLK from becoming cancerous lesions through the Connectivity Map database with molecular docking, providing a theoretical basis for the clinical diagnosis and treatment of OLK. The flow chart of this study is shown in Figure 1.

## 2. Materials and Methods

### 2.1. Microarray Chip Expression Data Source

The raw data of GSE85195 [10] and GSE25099 [11] were downloaded from Gene Expression Omnibus (https://www.ncbi.nlm.nih.gov/geo/) and processed using R software (4.0.5). Agilent's microarray sequencing chip was used in GSE85195; the sample type was *Homo sapiens* tissue samples; and the sequencing platform was the GPL6480 (Agilent-014850 Whole Human Genome Microarray 4 × 44 K G4112 F). The samples included 1 normal tissue, 15 OLK tissues, and 34 OSCC tissues. Affymetrix's microarray sequencing chip was used in GSE25099; the sample type was *Homo sapiens* tissue samples; and the sequencing platform was the GPL5175 (Affymetrix Human Exon 1.0 ST Array). The samples included 22 normal tissues and 57 OSCC tissues. The detailed information is shown in Table 1. The normalizeBetweenArrays function of the limma package [12] and the RMA method of the affy [13] package was used to perform data standardization, normalization, and gene annotation, remove probes without annotation information, take the average expression when the same probe appears multiple times, and take the common gene combined data in the two datasets. The ComBat method of the sva package [14] was used to remove batch effects between multiple datasets to obtain a gene expression matrix.

### 2.2. Weighted Gene Coexpression Network Analysis (WGCNA)

WGCNA package [15] was used to perform weighted gene coexpression network analysis: First, the hclust function was used to cluster the above gene expression matrix, remove outliers, and construct a gene relationship network for all the gene data in the remaining samples. The pickSoftThreshold function was used to select the best soft threshold. Then, the gene module was constructed, and blockwiseModules function was used to identify gene modules, set the minimum number of genes in the module to 20, the maximum number of genes to 7,000, and the tree cutting height value to 0.25. Finally, the correlation between each gene module and the clinical phenotype was calculated, moduleEigengenes function was used to calculate the module eigengene (ME) in each module, and the first principal component in the principal component analysis result was used to express the gene expression in each module. The overall level of the module's feature values was compared with the normal samples, OLK samples, OSCC samples, and status (status phenotype represented the dynamic process of progression from normal to OLK to OSCC). Pearson's correlation analysis was performed, the modules with the strongest correlation with the phenotype were selected, and the correlation coefficient was changed in sequence with the progression of the disease.

### 2.3. Functional Enrichment Analysis of Gene Modules

In order to dig deeper into the biological functions of gene modules, the abovementioned selected gene modules were subjected to Gene Ontology (GO) enrichment analysis and Kyoto Encyclopedia of Genes and Genome (KEGG) enrichment analysis, in the Database for Annotation, Visualization, and Integrated Discovery (David, https://david.ncifcrf.gov/Home.jsp). The biological processes, cell components, molecular functions, and KEGG pathways were selected, and the results were exported to obtain pathways related to OLK and OSCC.

### 2.4. The Protein Interaction Network Diagram of Gene Modules

The genes in the module with the strongest correlation with the clinical phenotype in the WGCNA results were extracted, and the STRING online database (https://string-db.org/) was used to score the genes in the module to predict the possibility of protein interactions and construct a protein-protein interaction network diagram. The more complex the structural relationship, the more important the core gene was in the development of the disease. The analysis results were imported into Cytoscape (v3.8.2), and the maximal clique centrality (MCC) algorithm in the cytoHubba plugin was used to mark the top 10 key genes.

### 2.5. Differential Expression Analysis

We compared the three groups of samples in the abovementioned high-throughput gene expression matrix pair by pair, and the limma package [12] in R software (4.0.5) was used for differential expression analysis. The calculation method of the difference between any two groups was that the latter was compared with the former. The ratio was the fold change (FC) value, and then, the logarithmic value based on 2 was used. This value was represented by log_2_FC. If the log_2_FC value is greater than 0, it means that the latter was higher than the former. It was an upregulated gene and was represented by red. Otherwise, it is downregulated gene and was indicated in blue. According to the FDR criterion proposed by Benjamini and Hochberg in 1995, the adjusted *p* value was calculated by multiplying the *p* value by the ranking of the gene in the total genes. In differential gene expression analysis, the difference was considered to be meaningful when the multiplicity of difference was greater than 2, filter |log_2_FC| greater than 1, *p* value less than 0.05, and adjusted *p* value less than 0.05 as filter conditions. The volcano map and heat map were drawn. R software was used to compare the upregulated genes and downregulated genes of the three groups of normal, OLK, and OSCC with the most clinically relevant gene modules and drew the Venn diagram to obtain the key genes. The key genes were used as a diagnostic model to identify whether OLK was cancerous.

### 2.6. Small-Molecule Drug Prediction

Connectivity Map (https://clue.io/) is a database of chemical reagent action expression profiles. Researchers can use gene expression profiles to match chemical drugs in the database. The degree of enrichment is expressed by scores. A positive number means that the chemical drug action is the same as the expression profile results. Negative numbers represent the opposite. The higher the score, the more similar and the better the prediction effect. The gene expression profile of OLK vs. OSCC was entered into the Query tool in the database. We entered the 150 upregulated genes and 150 downregulated genes with the largest fold difference, and the top 5 small-molecule chemical drugs with the highest scores in the results were selected to be molecularly docked with key genes.

### 2.7. Molecular Docking

The three-dimensional structure of the protein molecules was downloaded from the AlphaFold database (https://alphafold.ebi.ac.uk/), and the small-molecule drugs were downloaded from the PubChem database (https://pubchem.ncbi.nlm.nih.gov/). The AutoDock Vina 1.2.0 for molecular docking was used for analyzing the three-dimensional structure. The PyMOL 2.5.2 software was used to draw three-dimensional docking images, and the Discovery Studio 2021 software was used to draw two-dimensional docking images. It is generally believed that when the binding energy is less than −4.25 kcal/mol, there is a certain binding activity between the small-molecule drug and the protein. When the binding energy is less than −5.0 kcal/mol, it is considered that the two have good binding activity, and when the binding energy is less than −7.0 kcal/mol, it is considered that the two have strong binding activity.

### 2.8. Verification in the External Dataset

The OLK group and OSCC group were extracted in the dataset of this study, and the expression level and diagnostic efficacy of the diagnostic model were compared. Then, GSE26549 [16] was used to verify the diagnostic model. Affymetrix's microarray sequencing chip was used in GSE26549; the sample type was *Homo sapiens* tissue samples; and the sequencing platform was the GPL6244 (Affymetrix Human Gene 1.0 ST Array). The samples included 86 OLK tissues. The median follow-up time was 6.08 years, and 35 of the 86 patients developed OSCC over the course. R software (4.0.5) was used to compare the expression differences of four key genes in different pathological grades of OLK. Since the samples of the severe dysplasia were less than 3, 2 severe dysplasia samples and 1 sample without pathological type were not included in the study. But all samples are included in the comparison of hyperplasia and dysplasia. Then, the receiver operating characteristic curve (ROC) is analyzed, and the diagnostic performance of different diagnostic models in the dataset is compared. In order to dig deeper into the functions of the key genes, the Gene Expression Profiling Interactive Analysis (GEPIA, http://gepia.cancer-pku.cn/) database was used to calculate the overall survival rate and disease-free survival rate, and its expression characteristics were observed in tumors.

### 2.9. Protein Expression in Healthy and Tumor Tissues

The Human Protein Atlas (HPA, http://www.proteinatlas.org/) database provides multispecies, multitissue, and multisite immunohistochemical staining sections, including a large number of normal tissues and cancer tissues. It contains immunohistochemical staining results of a variety of proteins and currently contains more than 26,000 kinds of antibodies. It was used to compare the expression levels of key genes in the normal head and neck tissues and squamous cell carcinoma tissues.

## 3. Results

### 3.1. Removal of the Batch Effect of Gene Expression Profiling Chip

**Figure 2:**
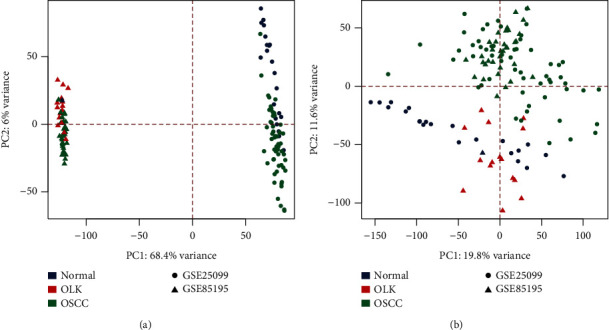
PCA before batch correction (a) and PCA after batch correction (b). The shapes represent different datasets, the circle is GSE25099, the triangle is GSE85195, the colors represent different tissue samples, dark blue represents normal tissue, red represents OLK tissue, and green represents OSCC tissue.

After the two datasets of GSE85195 and GSE25099 were merged, they contained 13,905 genes and 129 samples, including 23 normal tissue samples, 15 OLK samples, and 91 OSCC samples. There were no missing values in the gene expression matrix.  Figure 2 shows the principal component analysis (PCA) results before and after batch correction. Before the batch correction, the datasets were clustered and the datasets were separated, indicating that there was an obvious batch effect. After batch correction, the datasets were clustered and the tissue samples were separated, indicating that the batch effect had been removed and could be used for subsequent analysis.

### 3.2. Construction of Gene Coexpression Network

**Figure 3:**
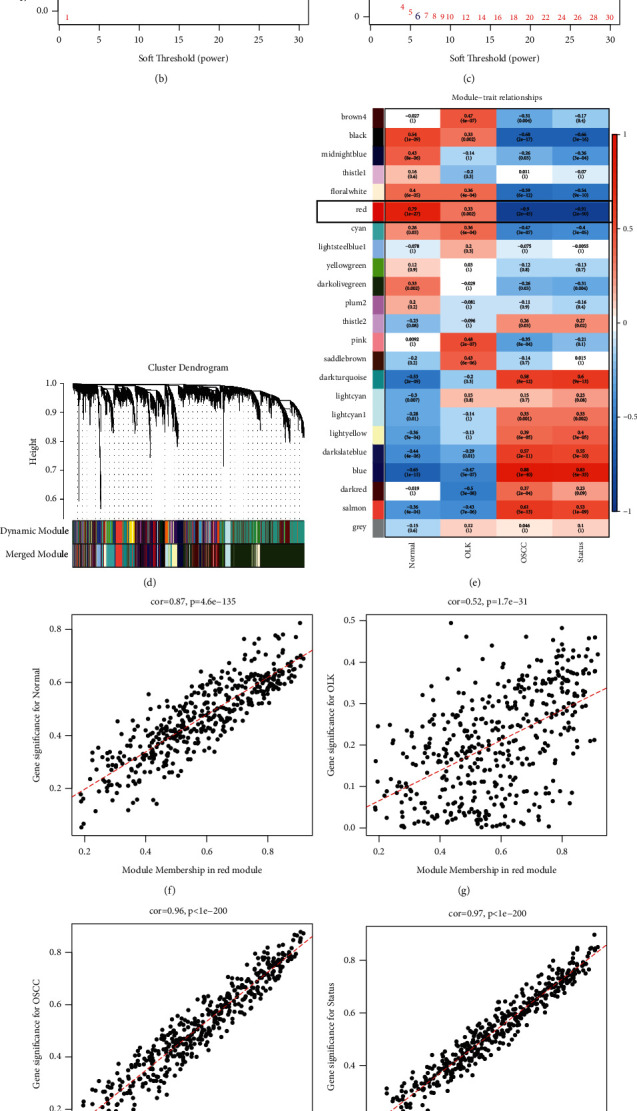
Sample clustering, and no outlier samples are generated (a). The relationship between the scale-free topology model fit and the soft threshold (b). The relationship between mean connectivity and soft threshold (c). Gene coexpression module construction, Dynamic Module is the result of the premerged module, and Merged Module is the result of the merged module (d). Heat map of the correlation between the module and the clinical phenotype (e), red represents positive correlation, and blue represents negative correlation. The relationship between the GS and the MM of the red module gene (f-i). The relationship of normal (f). The relationship of OLK (g). The relationship of OSCC (h). The relationship of status (i). The gene expression level of the module eigengene of the red module in each sample (j), the abscissa is the sample, the ordinate is the gene expression in the red module, red represents high expression, green represents low expression, the top is the heat map, and the bottom is each module eigengene of the red module in the sample.

We performed cluster analysis on all samples and found no outliers (Figure 3(a)), so all samples were included for subsequent analysis. First, we constructed a gene relationship network: when *R*^2^ is more than 0.8 and the average connectivity is less than 100 (Figures 3(b) and 3(c)), the best soft threshold was selected as 6. Then, we constructed the gene module according to the selection of the above soft threshold. When the branch height was less than 0.25, it was considered that the gene similarity exceeds 75% and could be merged into one module, and the genes that cannot match any module were merged into a gray module. A total of 23 modules were identified (Figure 3(d)).

Subsequently, cluster analysis was performed on all gene modules (Supplementary Figure 1A), and a correlation heat map was drawn (Supplementary Figure 1B). It was found that each gene module has little correlation with other modules and has a strong correlation with its own module. The adjacency relationship or topological overlap relationship was represented by a heat map (Supplementary Figure 2), and it was found that each gene had a strong correlation with the module gene. The correlation between the genes in the module was represented by a density map (Supplementary Figure 3). It was found that except for the gray module, the correlation coefficients of genes and modules in other modules were all basically distributed above 0.5, indicating that the genes in the module were highly correlated with each related module. These results suggested that the module construction was reasonable. Supplementary Figure 4 shows the relationship between module connectivity and genes. The connectivity of each gene in the module was the sum of its correlation with other genes. We found that except for the gray module, the greater the correlation coefficient between a gene and a module, the greater the mean connectivity of the gene, the more the gene was at the core of the module, and the network in each module conforms to the scale-free network.

Finally, the correlation between the gene module and the clinical phenotype was analyzed (Figure 3(e)). It was found that the red module had the strongest correlation with the disease development (*r* = −0.91, *p*=2*e* − 50), which contained a total of 435 genes. The correlation coefficient of modular genes gradually decreased with the progression of the disease. Figures 3(f)–3(i) show the correlation analysis of module membership (MM) and gene significance (GS) in the red module for the four phenotypes of normal, OLK, OSCC, and status. MM was the correlation between gene expression and module eigengene, and GS was the correlation coefficient between gene and phenotype. The correlation coefficient in the normal group was 0.87 (*p*=1.6*e* − 135), the correlation coefficient in the OLK group was 0.52 (*p*=1.7*e* − 31), the correlation coefficient in the OSCC group was 0.96 (*p* < 1*e* − 200), and the correlation coefficient in the status group was 0.97 (*p* < 1*e* − 200). We found that in the red module, MM and GS were related to the phenotype genes with stronger correlations that had higher module eigenvalues, and red modules were highly correlated with each phenotype. The expression trend of the red module gene in the sample showed that as the characteristic value of the module increases, the expression of the gene in the sample increases (Figure 3(j)), indicating that this module gene could be used to describe the sample.

### 3.3. Functional Enrichment Analysis

**Figure 4:**
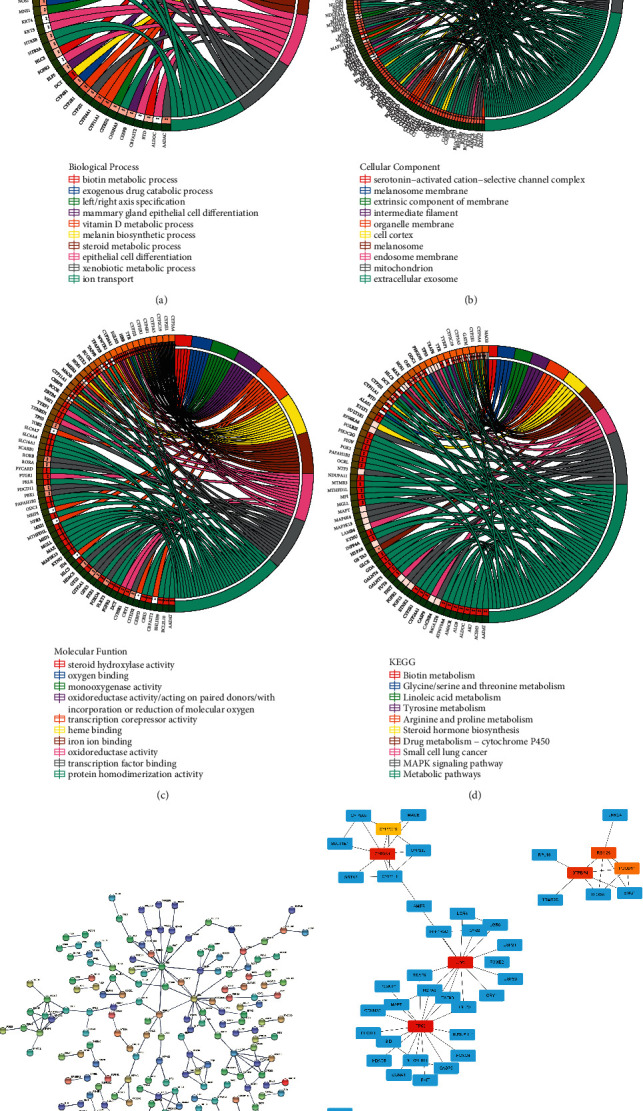
Circle graph shows the results of pathway enrichment analysis. The left half circle represents the gene, the right half circle represents the enriched pathway, and the two are connected by color bands (a-d). Biological process (a). Cellular component (b). Molecular function (c). KEGG pathway (d). Protein-protein interaction network (e), dots represent proteins, lines represent interactions, and the upper right corner is the gene encoding the protein. The top 10 core genes in the network (f). The darker the color, the more central the gene is in the network.

There were 60 GO analysis results, including 28 biological processes, 11 cell components, and 21 molecular functions (*p* < 0.05), sorted by *p* value from small to large.  Figure 4(a) shows the top 10 biological processes, which were mainly related to melanin biosynthetic process, vitamin D metabolic process, exogenous drug catabolic process, xenobiotic metabolic process, mammary gland epithelial cell differentiation, left/right axis specification, ion transport, steroid metabolic process, biotin metabolic process, and epithelial cell differentiation.  Figure 4(b) shows the first 10 cellular components, which were mainly related to melanosome membrane, melanosome, organelle membrane, cell cortex, extracellular exosome, endosome membrane, mitochondrion, intermediate filament, extrinsic component of membrane, and serotonin-activated cation-selective channel complex.  Figure 4(c) shows the first 10 molecular functions, which were mainly related to oxidoreductase activity/acting on paired donors/with the incorporation of or reduction in molecular oxygen, monooxygenase activity, heme binding, protein homodimerization activity, oxidoreductase activity, iron ion binding, steroid hydroxylase activity, oxygen binding, transcription factor binding, and transcription corepressor activity. There were 10 KEGG analysis results totally shown in Figure 4(d), which were mainly related to 4 statistically significant pathways. They were metabolic pathways, drug metabolism-cytochrome P450, linoleic acid metabolism, and arginine and proline metabolisms (*p* < 0.05).

### 3.4. Construction of a Protein Interaction Network Diagram

The genes in the red module were entered into the STRING database, *Homo sapiens* were selected as the species, and the noninteracting proteins were hidden. There were a total of 342 pairs of protein interactions. The network diagram formed is shown in Figure 4(e). The MCC algorithm in Cytoscape software could predict the protein or gene at the core position in the network and highlight the first 10 types. The darker the color indicated, the gene was more likely to be the core gene. These genes were as follows: TP53, UBC, CYP3A4, GTPBP4, RBM28, DCT, TYR, TYRP1, PDCD11, and CYP2C19 (Figure 4(f)).

### 3.5. Differential Expression Analysis

**Figure 5:**
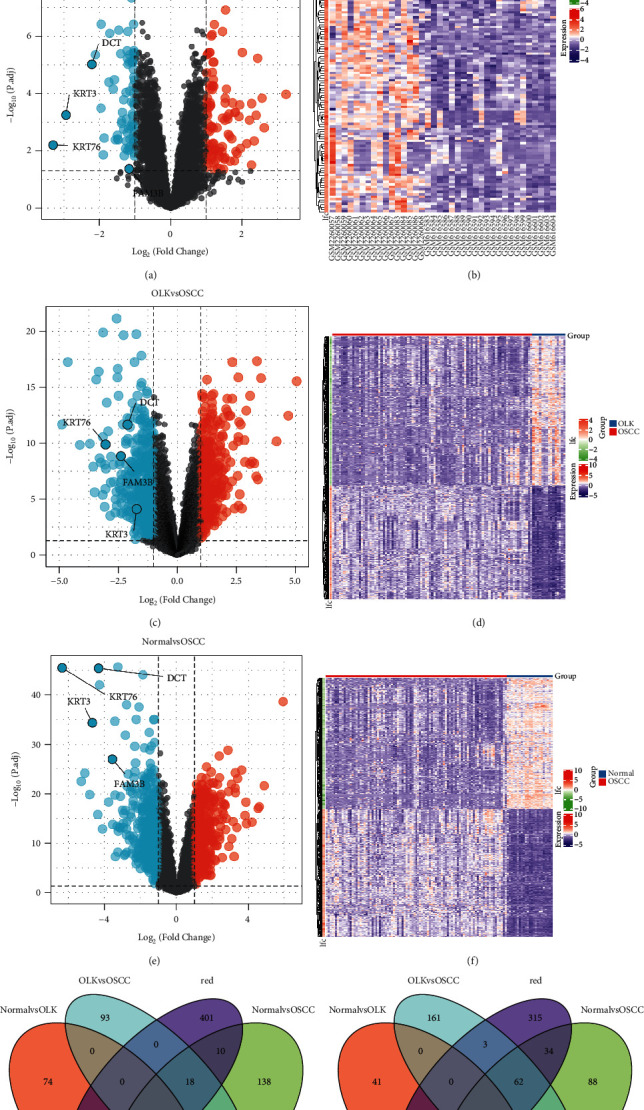
Volcano map of normal group vs. OLK group (a). The dotted line in the figure is the filter condition, black means that the difference is not obvious, red means upregulated genes, and blue means downregulated genes. Heat map of normal group vs. OLK group (b). Listed as samples, behavioral genes, red represents the OLK group, blue represents the normal group, red represents upregulation in the heat map, and dark blue represents downregulation. Volcano map of OLK group vs. OSCC group (c). Heat map of OLK group vs. OSCC group (d). Volcano map of normal group vs. OSCC group (e). Heat map of normal group vs. OSCC group (f). Intersection of differential genes in the healthy group, OLK group, and OSCC group compared with the genes in the red module (g-h). Upregulated genes (g). Downregulated genes (h).

The results of a pairwise comparison of the normal group, the OLK group, and the OSCC group showed that in comparing the normal group with the OSCC group, 176 genes were screened out according to the filter conditions, among them 101 genes were upregulated and 75 genes were downregulated (Figure 5(a) and 5(b)); in comparing the OLK group and the OSCC group, 780 genes were screened out, and among them, 337 genes were upregulated and 443 genes were downregulated (Figures 5(c) and 5(d)); in comparing the normal group with the OSCC group, 847 genes were screened out, and among them, 419 genes were upregulated and 428 genes were downregulated (Figures 5(e) and 5(f)). The results of the pairwise difference analysis among the normal group, the OLK group, and the OSCC group are intersected with the genes in the red module. There was no overlap in the upregulated genes, and there were 4 key genes in the downregulated genes, which were the dopachrome tautomerase (DCT) gene, keratin 3 (KRT3) gene, keratin 76 (KRT76) gene, and FAM3 metabolic regulation signal molecule B (FAM3B) gene (Figures 5(g) and 5(h)). These four key genes were marked in the volcano map.

### 3.6. Small-Molecule Drug Screening

The Connectivity Map database was used to compare and analyze the gene expression profile. We selected small-molecule drugs that could adjust the expression profile and ranked the top 5 according to the scoring from high to low (Table 2). The first place was selumetinib, which was a MEK inhibitor and MAP kinase inhibitor, with an enrichment score of −96.76. The second place was benidipine, which was a calcium channel blocker and L-type calcium channel blocker with an enrichment score of −95.74. The third place was levetiracetam, which was an acetylcholine receptor agonist, *N*-type calcium channel blocker, and synaptic vesicle glycoprotein ligand, with an enrichment score of −93.23. The fourth place was ampicillin, which was a cell wall synthesis inhibitor, with an enrichment score of −92.88. The fifth place was aminolevulinic acid, which was an oxidizing agent, with an enrichment score of −92.37.

### 3.7. Molecular Docking

**Figure 6:**
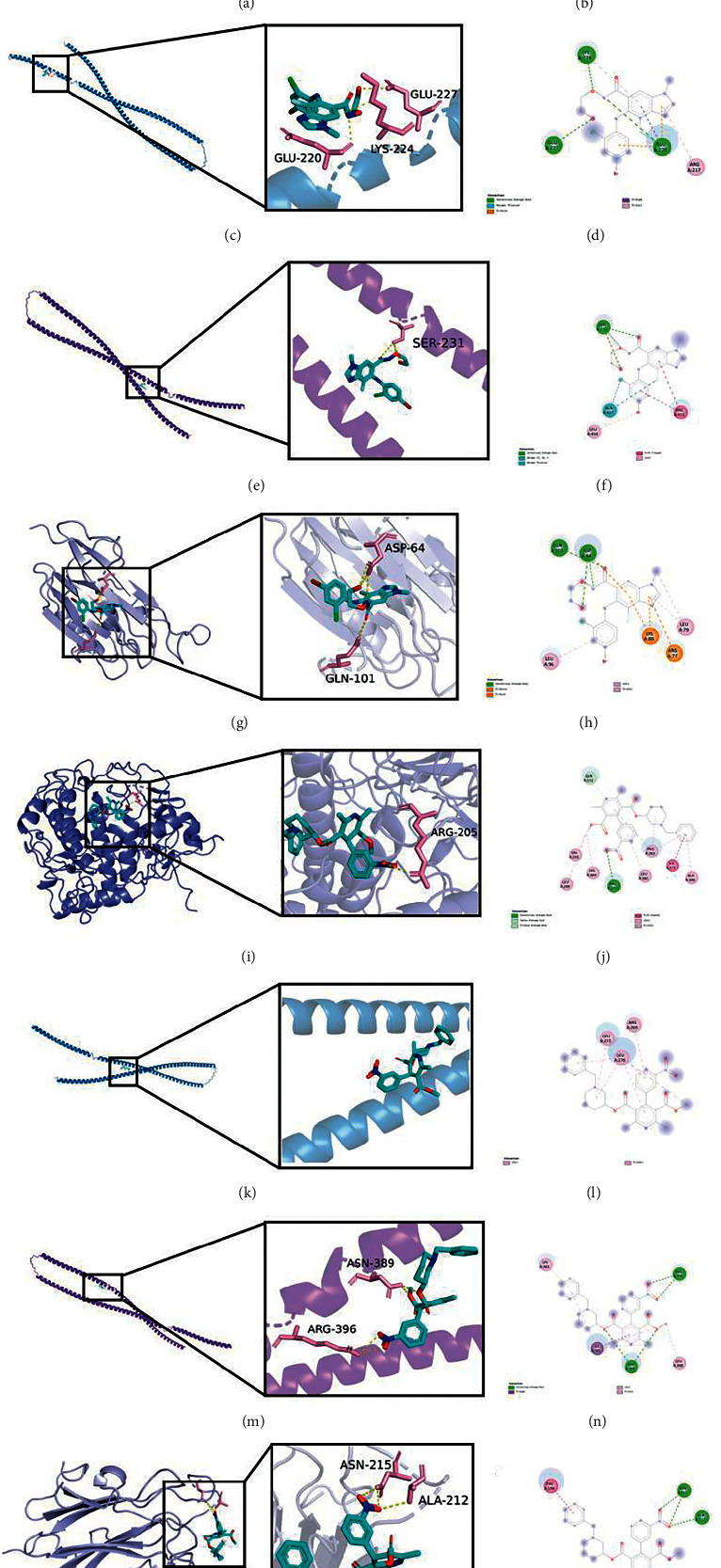
The three-dimensional view of the docking of selumetinib and DCT (a). Two-dimensional plan view of the docking of selumetinib and DCT (b). The three-dimensional view of the docking of selumetinib and KRT3 (c). Two-dimensional plan view of the docking of selumetinib and KRT3 (d). The three-dimensional view of the docking of selumetinib and KRT76 (e). Two-dimensional plan view of the docking of selumetinib and KRT76 (f). The three-dimensional view of the docking of selumetinib and FAM3B (g). Two-dimensional plan view of the docking of selumetinib and FAM3B (h). Three-dimensional view of the docking of benidipine with DCT (i). Two-dimensional plan view of the docking of benidipine with DCT (j). The three-dimensional view of the docking of benidipine and KRT3 (k). Two-dimensional plan view of the docking of benidipine and KRT3 (l). The three-dimensional view of the docking of benidipine and KRT76 (m). Two-dimensional plan view of the docking of benidipine and KRT76 (n). Three-dimensional view of the docking of benidipine and FAM3B (o). Two-dimensional plan view of the docking of benidipine and FAM3B (p).

We docked selumetinib and benidipine with the four molecules that were DCT, KRT3, KRT76, and FAM3B. The binding energy results of molecular docking are shown in Table 3. Figures 6(a) and 6(b) show the binding site maps of selumetinib and DCT. DCT formed hydrogen bond interactions with ligands through TYR-244, ASN-129, ASN-246, and ASP-458. The binding energy of selumetinib and DCT was −5.7 kcal/mol, showing a good binding activity. Figures 6(c) and 6(d) show the binding site map of selumetinib and KRT3. KRT3 formed hydrogen bond interactions with ligands through GLU-220, GLU-227, and LYS-224. The binding energy of selumetinib and KRT3 was -4.8 kcal/mol, showing a certain binding activity. Figures 6(e) and 6(f) show a map of the binding site of selumetinib and KRT76. KRT3 formed hydrogen bond interactions with ligands through SER-231. The binding energy of selumetinib and KRT76 was −4.6 kcal/mol, showing a certain binding activity. Figures 6(g)–6(h) show the binding site map of selumetinib and FAM3B. FAM3B interacted with the ligand through ASP-64 and GLN-101 to form a hydrogen bond. The binding energy of selumetinib and FAM3B was -5.6 kcal/mol, suggesting that they had a good binding activity.

The binding site map of benidipine and DCT is shown in Figures 6(i) and 6(j). DCT formed hydrogen bond interaction with ligand through ARG-205. The binding energy of benidipine and DCT was -8.2 kcal/mol, indicating a strong binding activity. Figures 6(k) and 6(l) show the binding site map of benidipine and KRT3. KRT3 did not form hydrogen bond interactions with ligands through its own residues, but the binding energy calculation showed that the binding energy of KRT3 and benidipine is -4.8 kcal/mol, suggesting a certain binding activity, and the binding of the two might be through other interactions. Figures 6(m) and 6(n) show a map of the binding site of benidipine and KRT76. KRT76 formed hydrogen bond interaction with ligand through ASN-389 and ARG-396. The binding energy of benidipine and KRT76 was −4.6 kcal/mol, indicating a good binding activity. Figures 6(o) and 6(p) show the binding site map of benidipine and FAM3B. It could be seen from the figure that FAM3B forms a hydrogen bond interaction with the ligand through ASN-215 and ALA-212. The binding energy of benidipine and FAM3B was −6 kcal/mol, showing a good binding activity.

### 3.8. Verification in the External Dataset

**Figure 7:**
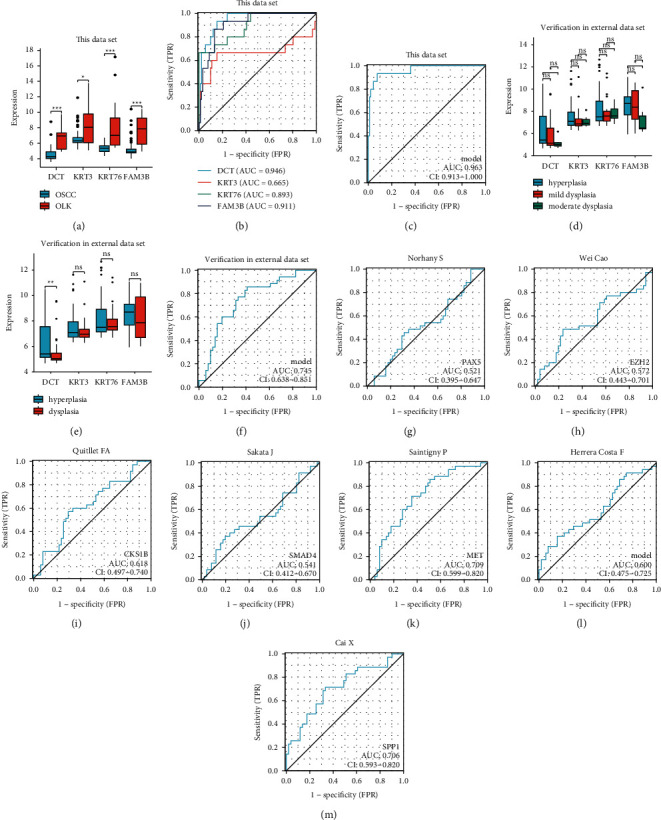
The expression levels of DCT, KRT3, KRT76, and FAM3B in this dataset (a). The diagnostic efficacy of DCT, KRT3, KRT76, and FAM3B in this dataset (b). The diagnostic efficacy of the combined diagnosis of four indicators (c). The difference between hyperplasia, mild dysplasia, and moderate dysplasia (d). The difference between hyperplasia and dysplasia (e). The diagnostic efficacy of the combined diagnosis of four indicators in the external dataset (f). Comparison of the previous diagnosis model and the diagnosis model of this study (g-m).

In the combined dataset of GSE85195 and GSE25099, the expression of DCT, KRT76, and FAM3B in the OSCC group was significantly lower than that of the OLK group, and the expression of KRT3 in the OSCC group was lower than that of the OLK group (*p* < 0.05) (Figure 7(a)). The ROC curve analysis showed that the area under the curve in which DCT, KRT3, KRT76, and FAM3B distinguished OLK from OSCC is 0.946 (CI = 0.900–0.991), 0.665 (CI = 0.451–0.880), 0.893 (CI = 0.802–0.984), and 0.911 (CI = 0.844–0.977), respectively (Figure 7(b)). When the four indicators jointly distinguished OLK from OSCC, the area under the curve was 0.963 (CI = 0.913–1.000), sensitivity 0.933, and specificity 0.923, which had high diagnostic accuracy (Figure 7(c)). The dataset of GSE26549 was downloaded to compare the differences between four key genes in different pathological grades of OLK and analyze the ROC curve of the diagnostic model in OLK and OSCC. The results showed that the difference between hyperplasia and moderate dysplasia of DCT and FAM3B was statistically significant (*p* < 0.05), and the differences in other genes among the three groups were not statistically significant (Figure 7(d)). The comparison of hyperplasia and dysplasia showed that the difference between the two groups of DCT was statistically significant (*p* < 0.05), and the difference of other genes between the three groups was not statistically significant (Figure 7(e)). The ROC curve analysis showed that the area under the curve that distinguished OLK from OSCC was 0.745 (CI = 0.638–0.851), which had a certain diagnostic accuracy (Figure 7(f)). By comparison, this diagnostic model had the highest diagnostic efficiency in previous studies [6, 7, 17–21] (Table 4), and the ROC curve is shown in Figures 7(g)–7(m).

**Figure 8:**
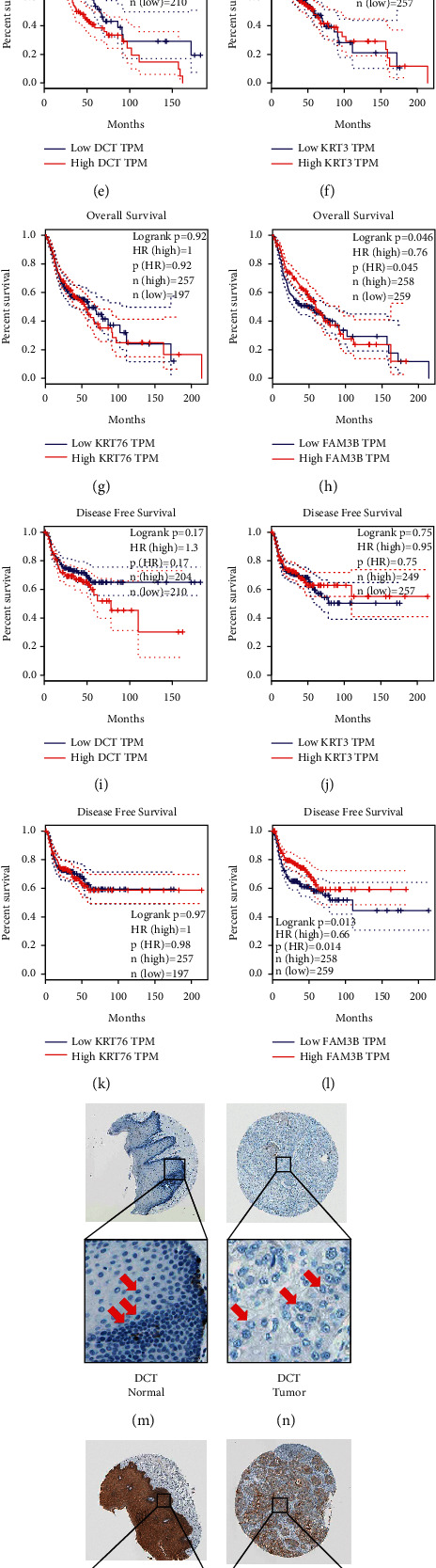
The expression of DCT in healthy tissues and tumor tissues throughout the body. Red represents tumor tissue, and black represents healthy tissue (a). The expression of KRT3 in healthy tissues and tumor tissues throughout the body (b). The expression of KRT76 in healthy tissues and tumor tissues throughout the body (c). The expression of FAM3B in healthy tissues and tumor tissues throughout the body (d). Survival curve of DCT gene overall survival rate (e). Survival curve of KRT3 gene overall survival rate (f). Survival curve of KRT76 gene overall survival rate (g). Survival curve of FAM3B gene overall survival rate (h). Survival curve of DCT gene disease-free survival rate (i). Survival curve of KRT3 gene disease-free survival rate (j). Survival curve of KRT76 gene disease-free survival rate (k). Survival curve of FAM3B gene disease-free survival rate (l). DCT expression in normal oral epithelial tissues (m). DCT expression in oral squamous cell carcinoma (n). Expression of KRT76 in normal oral epithelial tissues (o). Expression of KRT76 in oral squamous cell carcinoma (p). FAM3B expression in normal oral epithelial tissues (q). FAM3B expression in oral squamous cell carcinoma (r).

According to the pan-cancer study of the GEPIA database, DCT was highly expressed in skin cutaneous melanoma (SKCM) and almost not expressed in normal skin tissues and other tissues (Figure 8(a)). The expression of KRT3 was highest in the normal esophageal epithelium and oral mucosal epithelium of the head and neck. It was almost not expressed in other tissues of the body, and its expression decreased when it underwent cancerous transformation (Figure 8(b)). The expression of KRT76 was the highest in normal head and neck oral mucosal epithelial tissues, it was almost not expressed in other tissues of the body, and its expression was reduced when cancer occurs (Figure 8(c)). The expression of FAM3B in tumors of various systems was sometimes upregulated and sometimes downregulated, and its expression was decreased in head and neck squamous cell carcinoma (HNSC) (Figure 8(d)), which validated our results.

Survival analysis showed that there was a difference in the overall survival rate between the two groups with high and low expression of DCT gene (logrank *p*=0.04) (Figure 8(e)), and the high expression group was the risk group. The overall survival rate of the FAM3B gene high- and low-expression groups was different (logrank *p*=0.046) (Figure 8(h)), and the low-expression group was the high-risk group. There were also differences in the survival curves of disease-free survival between the two groups (logrank *p*=0.013) (Figure 8(l)). The low-expression group was the high-risk group, and the remaining survival curves had no statistical difference (Figures 8(f) and 8(g), and Figures 8(i)–8(k)).

### 3.9. Protein Expression in Normal and Tumor Tissues in the HPA Database

We searched the expression levels of four genes in normal oral epithelial tissues and tumor tissues, but KRT3 was not found in the database. HPA database showed that DCT expression was higher in normal oral epithelial cells than in cancerous cells (Figures 8(m) and 8(n)). The cytoplasmic/membranous expression of KRT76 in oral epithelial cells was high in normal epithelial tissues, and its expression was reduced in squamous cell carcinoma (Figures 8(o) and 8(p)). The cytoplasmic/membranous expression of FAM3B in oral epithelial cells was reduced when the epithelial tissue undergoes cancerous change (Figures 8(q) and 8(r)). The conclusion was consistent with our above research results.

## 4. Discussion

In this study, the multichip joint analysis solved the problem of insufficient sample size in oral cancer research. The gene changes in the process of phenotypic changes of epithelium from normal to abnormal hyperplasia and cancer were analyzed. The one-step analysis of WGCNA among the three groups of samples simplifies the analysis process and does not require pairwise comparison analysis, and the correlation analysis results with the clinical phenotype can be directly determined. The key genes screened by the intersection of the gene module with the strongest correlation of WGCNA and differential expression analysis not only retain the most relevant genes in the WGCNA results with clinical phenotype but also retain the different genes in the differential expression analysis results and save the step of subjectively screening and optimizing the analysis process. The four genes screened in this study are not only significantly different between OLK and OSCC, but DCT is also different in simple hyperplasia and dysplasia, and DCT and FAM3B are also different in hyperplasia and moderate dysplasia. Other genes are not significantly different in different pathological grades of OLK, and the reason may be due to the small sample size. Severe dysplasia is most prone to cancer, so severe dysplasia of OLK is extremely difficult to obtain, which has a certain impact on the results.

DCT, also named as tyrosinase-related protein 2 (tyrosine 2, TYR2), is one of the three important enzymes for melanin synthesis in the human body. The lack of DCT can cause albinism. Studies have shown that the risk of squamous cell carcinoma in patients with albinism is 1,000 times higher than that of the general population [22]. Cell experiments [23] and pan-cancer studies have shown that primary melanoma, metastatic melanoma, and nevus occur when DCT is overexpressed, and squamous cell carcinoma occurs when DCT is expressed at a low level. KRT3 and KRT76 are the skeletal components of cells, which are mainly distributed in the skin, mucous membrane, esophagus, and other areas with a high degree of keratinization, and their expression is significantly reduced when cancerous change occurs. KRT3 mutations are related to corneal dystrophy [24]. Immunohistochemistry and KRT76 knockout mouse experiments showed that the expression of KRT76 was downregulated in normal oral tissues, oral precancerous tissues, and oral cancer tissues [25]. The reason for increased tumor susceptibility is related to the change in the tumor microenvironment and immune factors [26]. FAM3B is a cytokine-like protein that regulates glucose and lipid metabolism by interacting with the liver and pancreas. Its elevated expression is related to type 2 diabetes, colon cancer, prostate cancer, and more. Staining of tissue sections showed that FAM3B expression decreased in OSCC tissues. Mouse experiments showed that knockdown of FAM3B promotes cell apoptosis by upregulating p53 in mice [27], and the expression of p53 protein increases in OSCC [28]. Therefore, FAM3B might cause OSCC by upregulating p53 protein.

GO enrichment analysis in the red module showed that OLK was related to the abnormal expression of multiple pathways, including vitamin D metabolic process, steroid metabolic process, exogenous drug catabolic process, and so on. Ras activation suppressed vitamin D transcriptional activity, vitamin D levels fell, and the risk of OLK cancer and OSCC increased. The prognosis and overall quality of life of patients with OSCC were affected by abnormal expression of the vitamin D metabolic pathway [29]. As a steroid hormone derivative, vitamin D also inhibited the activation of the NF-*κ*B pathway mediated by lcn2 via RPS3, enhancing the susceptibility of OSCC to cisplatin and the efficacy of treatment [30]. Patients with OSCC had considerably greater plasma and saliva cortisol levels than patients with OLK, patients with OLK had higher cortisol levels than normal people, and those with advanced stages had higher cortisol levels than those with early stages. As a result, OSCC is related to aberrant steroid metabolism [31]. Meanwhile, the drug efficacy of the patients with OSCC was influenced by the exogenous drug catabolic process. Endogenous and external triggers such as pH, matrix metalloproteinases (MMPs), reactive oxygen species (ROS), redox conditions, light, and magnetic fields could activate the stimulating response drug delivery system and improve the prognosis [32].

KEGG enrichment analysis in the red module showed that drug metabolism-cytochrome P450 and arginine, linoleic acid metabolism, and proline metabolism were related to OSCC. Cytochrome P450 2R1 (CYP2R1) is a vitamin D 25-hydroxylase that is involved in the conversion of dietary vitamin D to the active metabolite 25-(OH)-D_3_. CYP2R1 and vitamin D receptor (VDR) mRNA expression considerably rose in OSCC, according to the real-time RT-PCR study [33]. Simultaneously, the expression of the cytochrome P450 subtypes 1A1 and 1B1 (CYP1A1 and CYP1B1) genes increased in the head and neck cancer (HNC) cell line [34], elucidating the involvement of cytochrome P450 in OSCC. In the amino acid codon 72 of the p53 protein, there is a single-nucleotide polymorphism that encodes arginine (Arg) or proline (Pro). The arginine genotype of the OSCC group lowered the risk of oral cancer compared to the normal control group; however, the proline allele raised the risk of OSCC [35], showing the importance of arginine and proline metabolism in OSCC. The relevant results are consistent with the conclusions of this study.

OLK is an important step in the process of oral mucosal carcinogenesis, which requires long-term close monitoring and follow-up. The current monitoring of OLK is limited to clinical manifestations and lacks clear molecular indicators of cancer. The key genes screened in this study provide high-performance diagnostic indicators for the long-term monitoring of OLK and early diagnosis of OSCC. The genes DCT, KRT3, and FAM3B are reported for the first time in OSCC, and their expression remains to be evaluated. More samples are still needed to expand to verify the conclusions, and more intensive biological verification should be carried out in cell experiments and animal experiments.

## Figures and Tables

**Table 1:** Microarray chip expression data source.

Year	Series	Experiment type	Company	Platform	Sample type	Normal	OLK	OSCC
2017	GSE85195	Expression profiling by array	Agilent	GPL6480	Tissue	1	15	34
2011	GSE25099	Expression profiling by array	Affymetrix	GPL5175	Tissue	22	0	57
Total						23	15	91

OLK : oral leukoplakia; OSCC : oral squamous cell carcinoma.

**Table 2:** Small-molecule drugs from the connectivity map database.

Rank	ID	Type	Name	Description	Score
1	BRD-K57080016	CP	Selumetinib	MEK inhibitor and MAP kinase inhibitor	−96.76
2	BRD-A35519318	CP	Benidipine	Calcium channel blocker and L-type calcium channel blocker	−95.74
3	BRD-K49404994	CP	Levetiracetam	Acetylcholine receptor agonist and N-type calcium channel blocker, synaptic vesicle glycoprotein ligand	−93.23
4	BRD-A16754160	CP	Ampicillin	Cell wall synthesis inhibitor	−92.88
5	BRD-K57631554	CP	Aminolevulinic acid	Oxidizing agent	−92.37

ID : broad identity; CP: compound.

**Table 3:** Binding energy of molecular docking.

No.	Receptor	Ligand	Minimum (kcal/mol)	The average of the first five smallest values (kcal/mol)	Average of all minimums (kcal/mol)
vina1	DCT	Selumetinib	−5.7	−5.24	−5.03333
vina2	DCT	Benidipine	−8.2	−6.56	−5.95556
vina3	DCT	Levetiracetam	−5	−4.58	−4.25556
vina4	DCT	Ampicillin	−5.5	−5.46	−5.4
vina5	DCT	Aminolevulinic acid	−5.1	−4.7	−4.54444
vina6	KRT3	Selumetinib	−4.8	−4.46	−4.31111
vina7	KRT3	Benidipine	−4.8	−4.46	−4.3
vina8	KRT3	Levetiracetam	−3.5	−3.28	−3.15556
vina9	KRT3	Ampicillin	−4.8	−4.6	−4.53333
vina10	KRT3	Aminolevulinic-acid	−2.9	−2.86	−2.76667
vina11	KRT76	Selumetinib	−4.6	−4.44	−4.34444
vina12	KRT76	Benidipine	−4.6	−4.5	−4.38889
vina13	KRT76	Levetiracetam	−3.4	−3.12	−2.91111
vina14	KRT76	Ampicillin	−4.4	−4.12	−4.05556
vina15	KRT76	Aminolevulinic acid	−3	−2.78	−2.68889
vina16	FAM3B	Selumetinib	−5.6	−5.3	−5.11111
vina17	FAM3B	Benidipine	−6	−5.68	−5.44444
vina18	FAM3B	Levetiracetam	−4.4	−4.02	−3.73333
vina19	FAM3B	Ampicillin	−5.8	−5.48	−5.33333
vina20	FAM3B	Aminolevulinic acid	−3.8	−3.72	−3.54444

**Table 4:** Comparison of the previous diagnosis model and the diagnosis model of this study.

Year	Author	Molecular mark	AUC	CI
2021	Verification in external dataset	DCT, KRT3, KRT76, and FAM3B	0.745	0.638–0.851
2006	Norhany et al. [17]	PAX5	0.521	0.395–0.647
2011	Cao et al. [18]	EZH2	0.572	0.443–0.701
2011	Quitllet et al. [19]	CKS1B	0.618	0.497–0.740
2017	Sakata et al. [20]	SMAD4	0.541	0.412–0.670
2018	Saintigny et al. [21]	MET	0.709	0.599–0.820
2019	Herrera Costa et al. [7]	ALDH1A1 and ALDH1A2	0.600	0.475–0.725
2021	Cai et al. [6]	SPP1	0.706	0.593–0.820

AUC : area under the curve; CI : confidence interval.

## Data Availability

The data that support the findings of this study are openly available in GSE85195, GSE25099, and GSE26549 at https://www.ncbi.nlm.nih.gov/geo/.

## Supplementary Materials

Supplementary Materials Supplementary Figure 1: clustering tree between modules (A). Clustering heat map between modules (B). The abscissa and ordinate are the module names. Red is high similarity, and blue is low similarity. Supplementary Figure 2: the relationship between gene clusters and modules in each module. The darker the color, the stronger the correlation. Supplementary Figure 3: density plot of gene number and correlation coefficient. The abscissa is the correlation coefficient between genes and modules, and the ordinate is the number of genes. It is used to observe the distribution of correlation coefficients between each module and the gene expression in the module. A density map of 4 modules is drawn in each graph. Supplementary Figure 4: the relationship between connectivity and gene correlation in each module. The abscissa is the connectivity of each gene in each module, and the ordinate is the correlation coefficient between each gene in each module and the module.
