# Supplementary material for: Identification of Biomarkers Associated with Cancerous Change in Oral Leukoplakia Based on Integrated Transcriptome Analysis
Source: J Oncol. 2022 Jan 19;2022:4599305. doi: 10.1155/2022/4599305 (PMC8791753; doi:10.1155/2022/4599305)
Supplement: Supplementary Materials — Supplementary Figure 1: clustering tree between modules (A). Clustering heat map between modules (B). The abscissa and ordinate are the module names. Red is high similarity, and blue is low similarity. Supplementary Figure 2: the relationship between gene clusters and modules in each module. The darker the color, the stronger the correlation. Supplementary Figure 3: density plot of gene number and correlation coefficient. The abscissa is the correlation coefficient between genes and modules, and the ordinate is the number of genes. It is used to observe the distribution of correlation coefficients between each module and the gene expression in the module. A density map of 4 modules is drawn in each graph. Supplementary Figure 4: the relationship between connectivity and gene correlation in each module. The abscissa is the connectivity of each gene in each module, and the ordinate is the correlation coefficient between each gene in each module and the module. [file 4599305.f1.docx]

**Supplementary Figures**

| **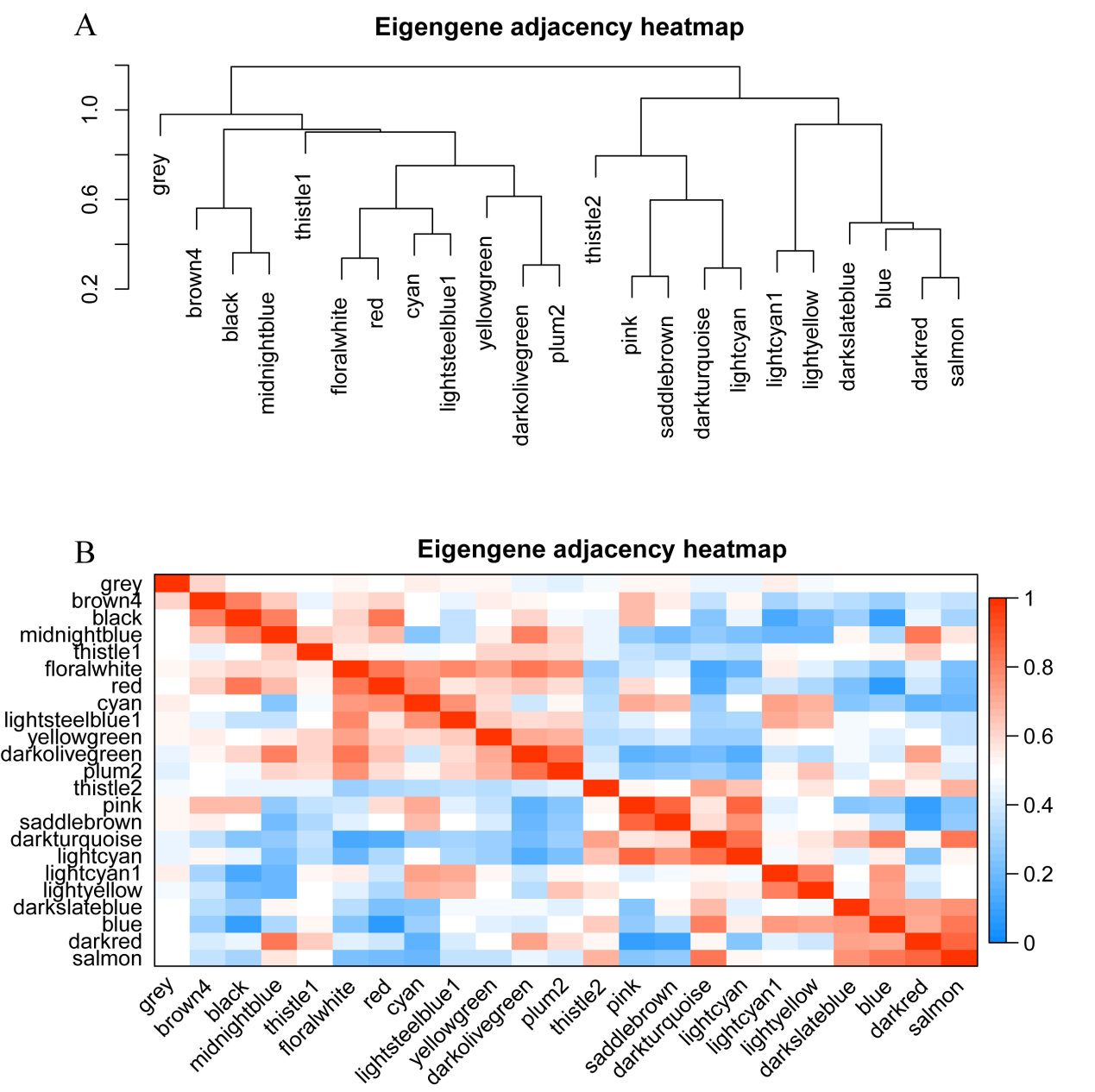** |
| --- |
| **Supplementary Figure 1** Clustering tree between modules (**A**). Clustering heat map between modules (**B**). The abscissa and ordinate are the module names. Red is high similarity, and blue is low similarity. |
| **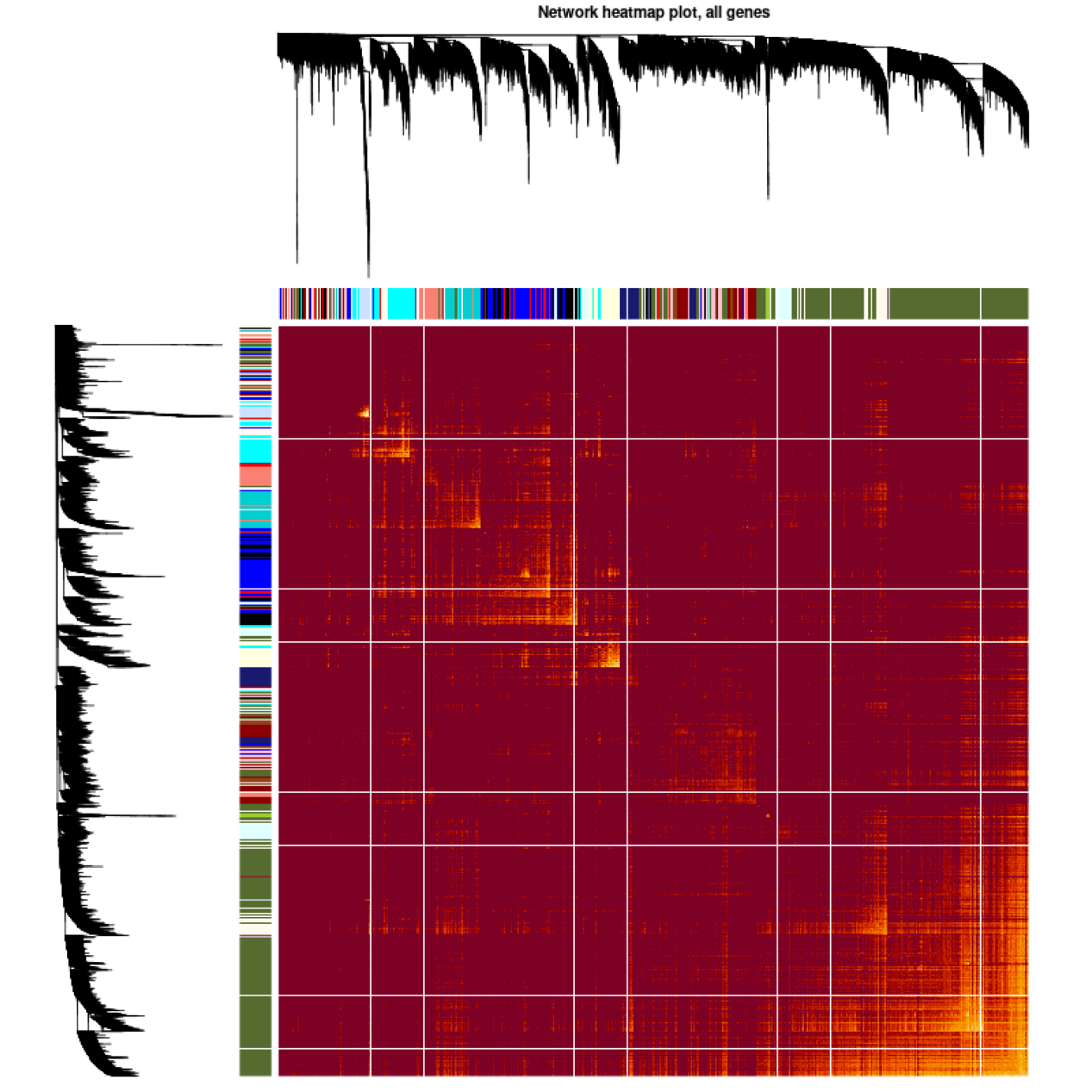** |
| **Supplementary Figure 2** The relationship between gene clusters and modules in each module. The darker the color, the stronger the correlation. |
| **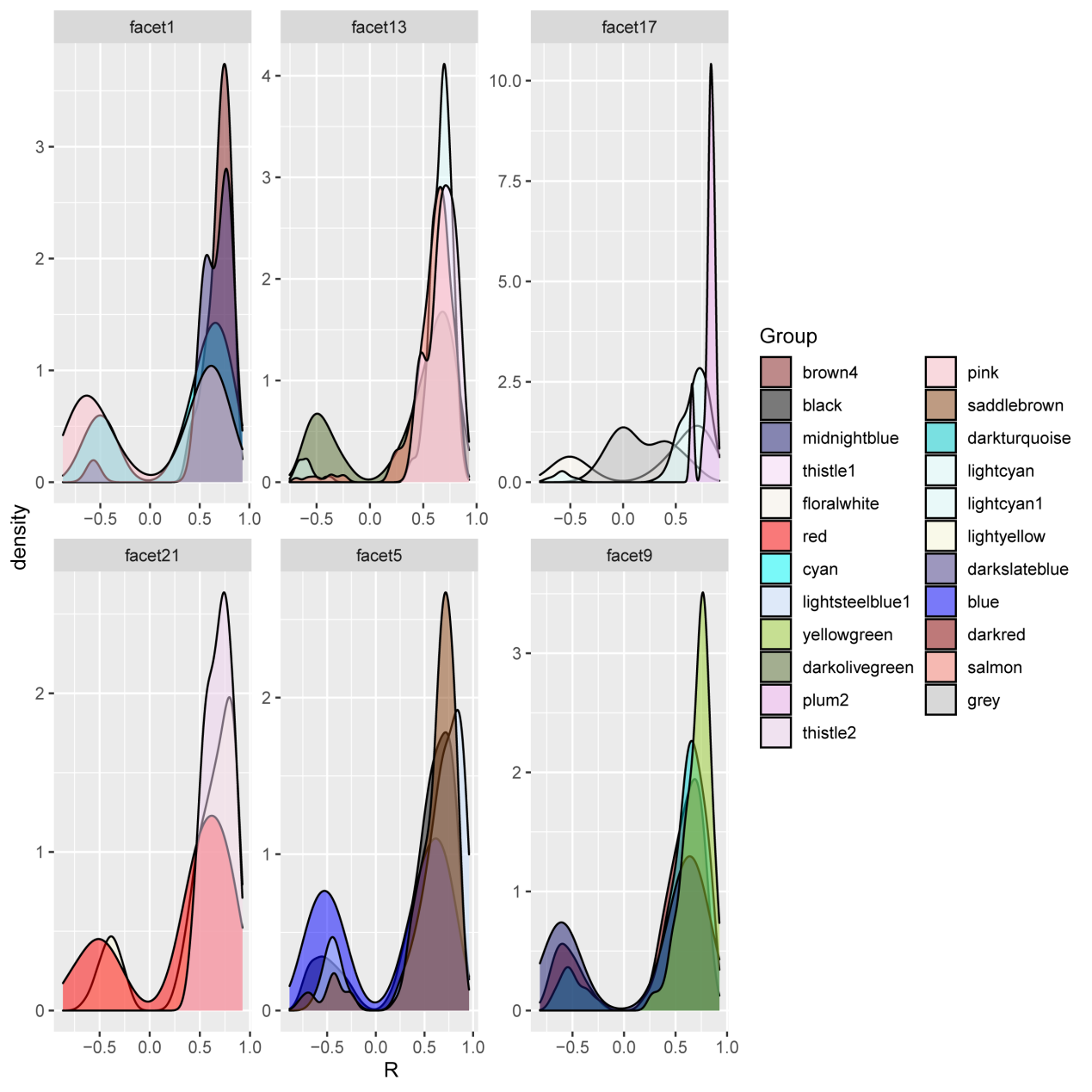** |
| **Supplementary Figure 3** Density plot of gene number and correlation coefficient. The abscissa is the correlation coefficient between genes and modules, and the ordinate is the number of genes. It is used to observe the distribution of correlation coefficients between each module and the gene expression in the module. A density map of 4 modules is drawn in each graph. |
| **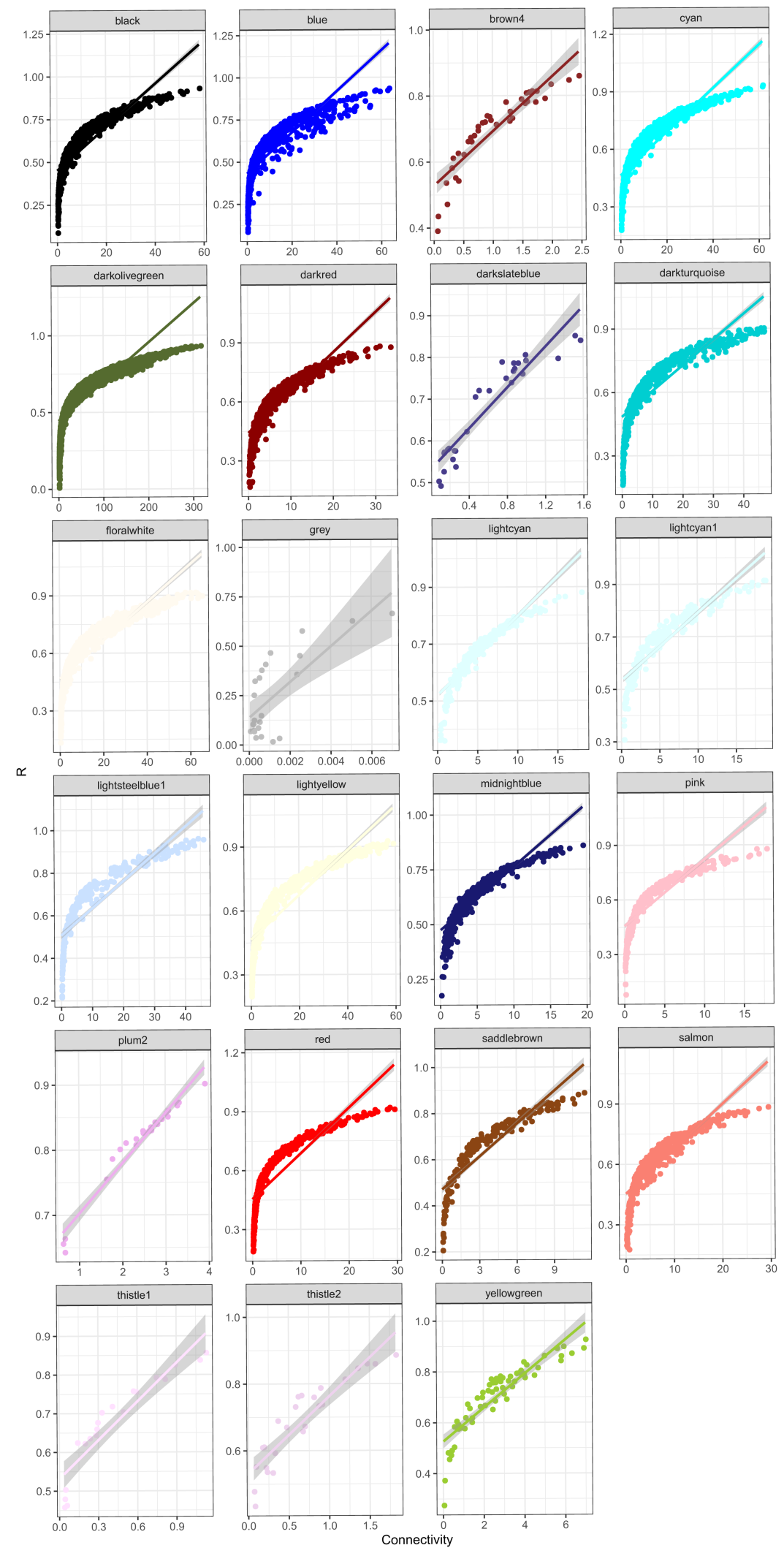** |
| **Supplementary Figure 4** The relationship between connectivity and gene correlation in each module. The abscissa is the connectivity of each gene in each module, and the ordinate is the correlation coefficient between each gene in each module and the module. |
